# Social innovation for health: engaging communities to address infectious diseases

**DOI:** 10.1186/s40249-020-00721-3

**Published:** 2020-07-18

**Authors:** Phyllis Dako-Gyeke, Uche V. Amazigo, Beatrice Halpaap, Lenore Manderson

**Affiliations:** 1 Department of Social and Behavioural Sciences, School of Public Health, University of Ghana, Accra, Ghana; 2 Pan-African Community Initiative on Education and Health (PACIEH), Enugu, Nigeria; 3 Special Programme for Research and Training in Tropical Diseases (UNICEF/UNDP/World Bank/WHO Special Programme for Research and Training in Tropical Diseases), Geneva, Switzerland; 4 School of Public Health, University of the Witwatersrand, Johannesburg, South Africa; 5 Institute at Brown for Environment and Society, Brown University, Providence, RI USA; 6 School of Social Sciences, Monash University, Melbourne, Australia

**Keywords:** Community engagement, Empowerment, Social innovation, Universal health coverage

## Abstract

Universal health coverage emphasises the value of the community-based delivery of health services to ensure that underserved populations have access to care. In areas where infectious diseases are endemic, there are often few resources and limited capacity, and the introduction of effective and accessible strategies require innovation. In this special issue, the contributing authors emphasise the power of local responses to the circumstances that underpin diseases of poverty, and highlight the methodological and programme innovations necessary to support and sustain these responses. Through case studies, the authors illustrate how social innovations can address health inequities, and they identify the role of academics in the Social Innovation in Health Initiative to support this approach.

## Background

The current goal of universal health coverage, and the challenges in implementing this through public sector programmes in resource-constrained settings, offer both urgency and opportunities for innovative approaches to address health inequities. In response, the Social Innovation in Health Initiative (SIHI) was launched in 2014 to advance understandings and the application of social innovations for community-based delivery of health services and local interventions for disease prevention and control in the global south [1, 2]. Leadership of this initiative was provided by the Special Programme for Research and Training in Tropical Diseases (TDR), together with the Bertha Centre for Social Innovation & Entrepreneurship at the University of Cape Town in South Africa, The Skoll Centre for Social Entrepreneurship at Oxford University, and the London School of Hygiene and Tropical Medicine.

Social innovation may be described as a collective process enabling the generation of ideas by people who participate collaboratively to achieve improved well-being [3]. The social objective behind this venture emphasises the engagement of concerned communities [3, 4]. This is particularly relevant to infectious diseases of poverty in endemic communities. Here community-directed programmes provide opportunities for government health services and other social actors, including non-government organisations and for-profit agencies, and individuals, to work closely with populations directly affected by such diseases. Mulgan and colleagues [5] emphasise that social innovation activities are predominantly developed with communities within which they are diffused, with innovative approaches meeting both social and medical needs. One such example is the community-directed intervention (CDI) approach, which has proven highly effective for mass drug administration in treating tropical diseases such as onchocerciasis and lymphatic filariasis [6, 7]. However, innovative CDI approaches have not yet been applied to scale up community education or health promotion, to address social determinants of diseases, or to establish community health insurance. These fields are at the core of Universal Health Coverage (UHC). There is a wide and growing gap between the scale of problems impacting underserved communities, and the scale and use of solutions known to emanate from these communities. This gap informs understandings of the challenges of UHC, as implied by the WHO definition, for sufficient, accessible, effective and quality services to be available without exposing the user to financial hardship. Social innovation and the inclusion of communities as key stakeholders in disease prevention and the promotion of community health insurance provide the means to improve health care delivery, achieve UHC and achieve the Sustainable Development Goals in low and middle income countries (LMICs).

## Social innovation and collaboration

Infectious diseases of poverty have immediate and long-term effects on individuals and communities, insofar as they are both caused by and compound other structural and institutional disadvantages and social inequalities (e.g. of gender, race and ethnicity), leading to stigma and social exclusion and negatively affecting cognitive development, literacy, economic activity and productivity. These impacts delay efforts towards community and national development and individual wellbeing [8, 9]. The acceleration and local adaptation of global strategies, to reduce infectious diseases of poverty by empowering people and communities to address their own health problems, provides an opportunity to tackle the interlocking challenges of disease, structural vulnerability, poverty and health systems limitations. The support of social innovations in health is one way to meet these challenges [2].

The Bertha Centre for Social Innovation & Entrepreneurship, in the Business School of the University of Cape Town, South Africa, has an established history in developing social innovations, and the establishment of SIHI within TDR drew on and found inspiration in this experience. In this special issue, de Villers and Bonnici describe how the Bertha Centre and other institutions work in different African settings towards social justice and to ensure social impact by applying innovative models for finance, health, education, and youth development. Their examples include the Transnet Phelophepa Health Trains, which use South Africa’s railway network to take model clinics into the country’s heartlands; the Pelebox Smart Locker for accessing TB treatment in Gauteng Province; and Last Mile Health in Liberia. In this latter example, through partnership with the Liberian government, Last Mile Health trains community health workers to use smartphone technology to prevent, diagnose and treat a range of medical conditions and diseases. By recruiting and equipping community members to deliver health services to neighbours, the government ensures that patients are able to access care from a trusted health professional, who they believe will provide them with the appropriate treatment and medication for common conditions such as malaria. This approach evidently overcomes challenges at a local level associated with illiteracy, social exclusion and social divisions, and promotes community trust in health care delivery.

While social innovation in the delivery of services occurs everywhere, many promising ideas in LMICs fail at the design stage due to the limited use of scientific approaches that enable institutional support and scale-up. The failure to generate compelling evidence for innovation derives from the lack of support for bottom-up approaches, in which context community members are involved in identifying local innovations. Crowdsourcing, as discussed in this issue, enables individuals and groups to propose a range of approaches and solutions to problems to be shared with the public. Support is critical for sustainability and the extension of innovative solutions.

Crowdsourcing provides opportunities to solicit social innovations, generate ideas and identify examples of individual entrepreneurship, but in LMICs there is limited experience of social innovations that have been developed and implemented for any purpose. To overcome this challenge, Tucker and colleagues have demonstrated how a crowdsourcing “designathon” can be used to design a public health programme [10]. This is a new model for multi-sectoral collaboration and community engagement. This methodological approach includes face-to-face meetings as well as internet engagement, thereby minimising concerns about limited internet access which in some LMICs might inhibit the engagement of sectors of the population.

Another model proposed by researchers is the engagement of university-based SIH hubs as cross-disciplinary and cross-sectoral platforms, which can catalyze the adoption of social innovation programmes. As van Niekerk and colleagues illustrate in this issue, these hubs were developed to engage with national and regional health systems and programmes through local research projects, community building, storytelling and institutional embedding. The authors argue that such hubs can work to build the necessary evidence to disseminate and institutionalise this approach, serve as a cross-disciplinary platform, promote SIH, and support SIH’s integration within national structures.

Positive and sustained changes resulting from social innovative approaches depend on interactions between the innovators and the environment, either within small local communities or in larger regional areas [3], and on community commitment and investment in such programmes. Although van Stam [11] proposed a three-step community engagement process for social innovation, the duration for local adaptation of a given project or approach often depends on the specific intervention being delivered within a community setting. Initial sensitisation about a given health condition and awareness creation within communities, empowerment of local stakeholders, and subsequent skills development, community education and implementation, can be particularly complex for preventive efforts. In contrast, interventions related to logistics, such as improved mechanisms for drug distribution or the delivery of pathology results, may move faster. For example, the Riders for Health initiative, which began operating in Lesotho and extended to Liberia, Kenya, Zimbabwe, Zambia, Malawi, the Gambia and Nigeria, required government support but was not dependent on community engagement for operation and success (https://socialinnovationinhealth.org/case-studies/riders-for-health-2/). Accordingly in this case community engagement followed from the success of the intervention, rather than being essential to its introduction.

In contrast, the programme described by Diana Castro-Arroyave and colleagues to reduce the transmission of Chagas disease, in this special issue, derives from community concern to tackle the infestation of triatomid bugs. The intervention required community knowledge of the epidemiology of the disease firstly, and then the willingness of individuals and households to commit resources, time and skills for housing improvement. Community sensitisation, engagement and empowerment are therefore critical for specific interventions and social innovation activities.

Social innovation indicators to monitor community engagement and action for health — the demand side of UHC - are also necessary in designing effective health services delivery. There is little consensus on how to define social innovation, its preconditions and dimensions, or on what circumstances ensure feasibility, scale-up and sustainability. Consequently, the development of indicators for measurement is complex and experimental [12, 13]. However, the use of four fundamental considerations in social innovation (i.e. people, the challenge, the process and the goal) is a credible and effective way to assess the extent of community engagement [3]. For infectious diseases, the innovation needs not only to seek to address the condition, but also to be closely linked with the processes of the inclusion of the community and the methods by which a specific challenge is understood and negotiated [3]. Although a framework may exist for engaging community members in social innovation, the specific methods adopted need to reflect and be acceptable within the local context, culture and politics. The actual processes therefore may also be a means to improve social circumstances by addressing domestic or environmental goals, with very conscious and continuous engagement with the community of interest.

Rhule and Allotey explore the features of social innovation and present and analyse the challenges of undertaking research within the context of community-driven innovation. They posit that research needs to be in the service of the community, and for this reason it requires innovation in approach and design to balance rigour with the realities of working with and responding to community driven demand. The authors also state that social innovation accommodates bottom up endeavours and community mobilisation as a form of activism. The core objective of community engagement is attainable, sustainable and replicable activities, and this influences how given projects, activities and/or services are accepted and adopted [11].

The development and introduction of social innovation will inevitably require iteration to review, evaluate and tailor activities to fit with any given community, and to continuously share knowledge, evolve perspectives and ensure ongoing interactions between communities and other stakeholders [3]. This process is aptly demonstrated in this issue by Castro-Arroyave and colleagues, whose example of the integrated control of Chagas disease illustrates how social innovation can generate processes of transformation in health while taking into account socio-cultural and economic conditions. The person or organisation initiating the innovation must work closely with local community members to establish necessary infrastructure as well as implement the interventions. This gradual and grounded process can be time consuming, especially when the innovator seeks to ensure maximum local community adoption, viability and sustainability.

## Conclusions

Endemic countries face challenges associated with the continued incidence of infectious diseases of poverty and other persistent health problems, constraints in infrastructure, resources and the accessibility of health services, and the limited capacity of local actors to identify appropriate, sustainable and scalable interventions. Social Innovation in Health as an initiative offers a means by which universities, agencies and government institutions can nurture, encourage and support local responses to everyday needs and constraints to access health services and intervene in disease transmission. As the articles in this issue illustrate, the institutionalisation of this approach aims to encourage an openness within state structures and by state actors to build local capacity, to learn from local experiences and circumstances, and to access resources. The approach allows academics and employees in government, among others, to learn from local populations new ways to respond to what might appear to be intractable problems. The approach encourages academics to work with communities, community-based organisations and local enterprise. The resultant collaborations will enable academics and government staff to gain familiarity with and generate the evidence of the effectiveness of local innovations, so to support their uptake, dissemination and sustainability.

## Data Availability

Not applicable.
